# The Role of Hydrogen Sulfide in the Localization and Structural–Functional Organization of p53 Following Traumatic Brain Injury: Development of a YOLO Model for Detection and Quantification of Apoptotic Nuclei

**DOI:** 10.3390/ijms26115066

**Published:** 2025-05-24

**Authors:** Evgeniya Kirichenko, Stanislav Bachurin, Anton Lisovin, Rozaliia Nabiullina, Marya Kaplya, Aleksandr Romanov, Chizaram Nwosu, Stanislav Rodkin

**Affiliations:** Research Laboratory “Medical Digital Images Based on the Basic Model”, Department of Bioengineering, Faculty of Bioengineering and Veterinary Medicine, Don State Technical University, 344000 Rostov-on-Don, Russia

**Keywords:** hydrogen sulfide, p53, traumatic brain injury, apoptosis, neurons, glial cells, YOLO

## Abstract

Traumatic brain injury (TBI) triggers a cascade of molecular and cellular disturbances, including apoptosis, inflammation, and destabilization of neuronal connections. The transcription factor p53 plays a pivotal role in regulating cell fate following brain injury by initiating pro-apoptotic signaling cascades. Hydrogen sulfide (H_2_S) may significantly contribute to the regulation of p53. Using scanning laser confocal microscopy, we found that after TBI, p53 accumulates extensively in the damaged cerebral cortex, showing distinct subcellular localization in neurons and astrocytes. In neurons, p53 predominantly localizes to the cytoplasm, suggesting involvement in mitochondria-dependent apoptosis, whereas in astrocytes, p53 is found in both the nucleus and cytoplasm, indicating possible activation of transcription-dependent apoptotic pathways. Quantitative analysis confirmed a correlation between p53 localization and morphological signs of cell death, as revealed by Sytox Green and Hoechst nuclear staining. Modulating H_2_S levels exerted a marked influence on p53 expression and distribution. Administration of the H_2_S donor sodium thiosulfate (Na_2_S_2_O_3_) reduced the overall number of p53-positive cells, decreased nuclear localization, and lowered the level of apoptosis. Conversely, inhibition of H_2_S synthesis using aminooxyacetic acid (AOAA) led to enhanced p53 expression, increased numbers of cells exhibiting nuclear fragmentation, and a more pronounced apoptotic response. These findings highlight a neuroprotective role for H_2_S, likely mediated through the suppression of p53-dependent cell death pathways. To improve analytical accuracy, we developed a YOLO-based deep-learning model for the automated detection of fragmented nuclei. Additionally, evolutionary and molecular dynamics analysis revealed a high degree of p53 conservation among vertebrates and indicated that, although H_2_S does not form stable complexes with p53, it may modulate its conformational dynamics.

## 1. Introduction

Traumatic brain injury (TBI) remains one of the leading causes of disability and mortality, particularly among young individuals. The pathogenesis of TBI involves key processes such as oxidative stress, inflammation, apoptosis, and neuroglial dysfunction, all contributing to secondary damage of neural tissue. A major challenge in modern neurobiology is to identify molecular mechanisms that can modulate these processes and reduce neuronal death [1,2]. One promising candidate is H_2_S, which has recently gained attention for its neuroprotective properties [3].

Hydrogen sulfide (H_2_S) is a gaseous transmitter actively synthesized in tissues and involved in a wide range of biological functions, including vascular tone regulation, neuromodulation, cytoprotection, inflammation, and apoptosis. Due to its volatility and low molecular weight, H_2_S can easily penetrate biological membranes and interact with intracellular targets [4,5,6,7,8]. While changes in brain H_2_S levels following TBI are known, the precise mechanisms by which it influences cellular regulatory systems remain insufficiently understood.

Of particular interest is the ability of H_2_S to modulate the activity of p53, a key tumor suppressor protein involved in cell cycle regulation, apoptosis, and DNA repair. Under normal conditions, p53 is maintained at low levels via ubiquitin-mediated degradation. Upon cellular stress or injury, p53 is stabilized and activates signaling pathways leading to genomic repair or programmed cell death. Studies suggest that H_2_S may regulate p53 both indirectly—by reducing oxidative stress and enhancing antioxidant enzyme activity; and directly—through S-sulfhydration of cysteine residues, altering the protein’s conformation and functional activity [9,10]. Interestingly, even weak van der Waals interactions can induce significant structural rearrangements in p53 [11,12], though these mechanisms remain poorly explored.

In parallel, automated detection of neural cells in fluorescence microscopy images has become a cornerstone of modern biomedical research, particularly in cellular and molecular neurobiology [13,14,15,16]. Recent advances in deep learning-based object detection have significantly accelerated the analysis of biological structures in images. A key role in this advancement has been played by You Only Look Once (YOLO) models developed by Ultralytics, which have become essential tools for automated image analysis [17]. YOLOv8, for instance, enables real-time object detection with high accuracy [18], and the recently released YOLOv11 introduces architectural improvements for better feature extraction and faster processing [19].

In our previous work, we thoroughly investigated the role of H_2_S in regulating p53 expression and its subcellular localization, as well as its impact on neuronal and glial cell death following TBI and axotomy. However, this earlier study utilized a non-invasive, moderate-severity TBI model and a different H_2_S donor, which limited its clinical relevance for severe TBI [20]. In the present study, we addressed this gap by employing a custom-developed severe penetrating TBI model that more accurately reflects the pathophysiological conditions of neural tissue under traumatic injury.

To further enhance the translational potential of our findings, we used sodium thiosulfate (Na_2_S_2_O_3_) as the H_2_S donor, a compound already approved for clinical use, including as an antidote for cyanide poisoning and as a therapeutic agent in conditions associated with oxidative stress. This choice aligns our experimental approach with clinically relevant therapeutic strategies, providing a more direct path to potential clinical applications.

Additionally, building on our expertise in automated image analysis [21], we developed and trained a YOLO-based neural network model for automated detection and quantification of apoptotic nuclei. This model was further integrated into a Telegram bot for high-throughput real-time image analysis, enabling rapid and standardized assessment of cellular damage. This combination of advanced computational tools and the severe TBI model represents a significant methodological advancement, offering a more precise and scalable approach for evaluating neural tissue damage.

The main goals of this study were to assess the effects of severe TBI on p53 expression and nuclear fragmentation in neural tissue; to explore the interactions between H_2_S and p53 at both molecular and cellular levels, including the evolutionary aspects of this process; and to evaluate the effectiveness of automated image analysis using the newly developed YOLOv11 model for detecting apoptotic changes in neural tissue.

Based on the results obtained, we formulated the central hypothesis that H_2_S-dependent signaling pathways play a critical role in the apoptotic cell death of neural cells following severe TBI, potentially through cytoplasmic–nuclear regulation of p53 expression, possibly involving direct interactions between H_2_S and p53 near the critical arginine 248 site (Arg248). Notably, neutral H_2_S demonstrated a weak but statistically significant reduction in binding energy, particularly near the Arg248 site, while the anionic form, HS^−^, showed minimal effect, consistent with the pH-dependent speciation of H_2_S under physiological conditions. This suggests that in acidic environments, such as those found in TBI, an increased proportion of neutral H_2_S may facilitate transient interactions with p53, potentially altering its transcriptional activity and contributing to neuroprotection.

Furthermore, it is proposed that automated image analysis using the YOLOv11 model can provide a standardized and efficient approach for assessing nuclear fragmentation, thereby improving the accuracy and reproducibility of morphological studies in TBI research.

## 2. Results

### 2.1. Spatial Analysis of p53 Distribution

**Figure 1:**
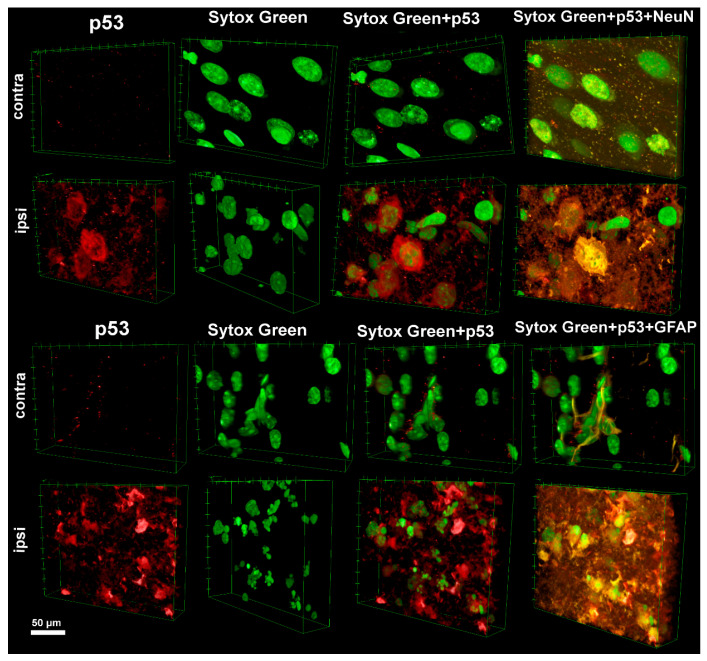
Confocal laser scanning microscopy, demonstrating p53 expression and nuclear morphology in the contralateral and ipsilateral hemispheres of the brain after TBI. Red signal corresponds to p53 immunolabeling, green to Sytox Green nuclear staining. Yellow signal indicates colocalization of Sytox Green with neuronal (NeuN) or astrocytic (GFAP) markers, visualizing cells with signs of degeneration. Scale bar: 50 μm.

Three-dimensional analysis of the spatial distribution of p53 in the contralateral (intact) and ipsilateral (injured) hemispheres of the brain after TBI revealed pronounced differences in its expression and intracellular localization. In the contralateral hemisphere, which did not undergo direct mechanical impact, confocal laser scanning microscopy combined with immunofluorescent staining for p53, the neuronal marker neuronal nuclei (NeuN), and the glial marker glial fibrillary acidic protein (GFAP) demonstrated minimal expression of p53. The protein was visualized as sparse, weak signals evenly distributed in the cytoplasm of individual neurons and astrocytes, with no signs of nuclear accumulation. Sytox Green staining, which is sensitive to structural changes in deoxyribonucleic acid (DNA), confirmed the absence of significant pathology: cell nuclei retained their characteristic round shape, smooth contours, uniform chromatin distribution, and intact nuclear membrane (Figure 1).

In contrast, the ipsilateral hemisphere showed a marked activation of p53, correlating with the area of traumatic damage. Three-dimensional reconstruction of confocal Z-stacks revealed intense accumulation of p53 in neurons and astrocytes, although its intracellular distribution differed significantly between these cell types. In neurons, p53 was predominantly localized in the cytoplasm, partially forming aggregates, whereas the nuclear signal was notably weaker. In astrocytes, p53 was detected in both the cytoplasm and nuclei (Figure 1).

p53 expression in the ipsilateral hemisphere was observed in cells exhibiting signs of apoptotic death. In both neurons and astrocytes, p53 activation was accompanied by pronounced chromatin condensation, visualized with Sytox Green staining as bright compact clumps, nuclear shrinkage, membrane blebbing on the nuclear envelope surface, and fragmentation of nuclei into apoptotic bodies (Figure 1).

Moreover, 3D image analysis showed that neurons with strong cytoplasmic p53 localization more frequently exhibited early stages of apoptosis, characterized by partial chromatin condensation. In astrocytes, in contrast, high nuclear expression of p53 was associated with active nuclear fragmentation into apoptotic bodies (Figure 1).

### 2.2. Fluorescence Microscopy of p53 Localization in Brain Neural Tissue After TBI

**Figure 2:**
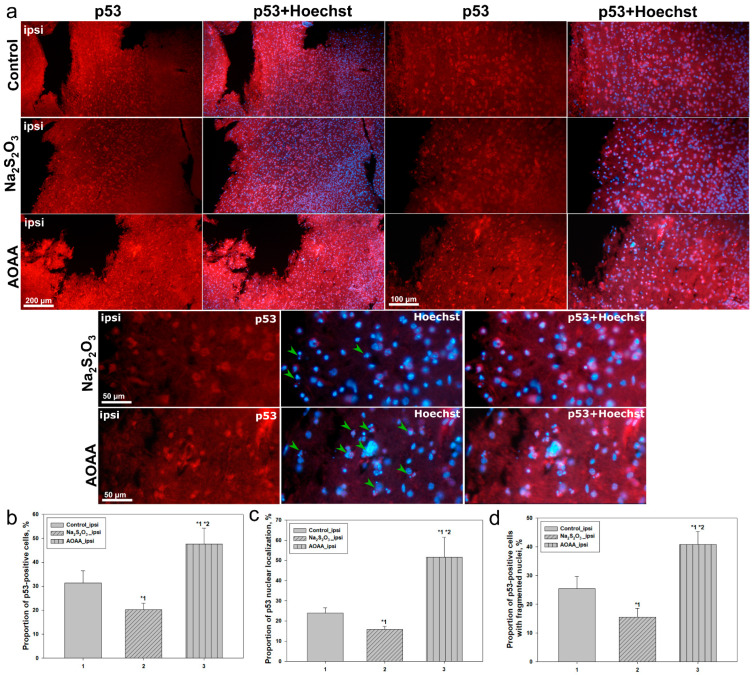
Fluorescence microscopy of the role of H_2_S in p53 localization in brain neural tissue after TBI. (**a**) Representative fluorescence microscopy images showing p53 (red) expression in the ipsilateral cortex in control animals and those treated with Na_2_S_2_O_3_ or AOAA. Nuclear staining was performed using Hoechst 33342 (blue), and p53 is visualized in the green channel. Green arrows indicate fragmented nuclei. (**b**) Quantitative graph showing the proportion of p53-positive cells relative to the total number of cells. (**c**) Graph showing the percentage of p53-positive cells with nuclear localization of p53 among all p53-expressing cells. (**d**) Graph of the number of p53-positive cells with fragmented nuclei among all p53-positive cells. * *p* < 0.05—comparisons between groups. The numbers below the bars correspond to group labels (1: Control_ipsi, 2: Na_2_S_2_O_3__ipsi, 3: AOAA_ipsi), while the significance markers (*1, *2) above the bars indicate statistically significant differences between the respective groups. Data are presented as mean ± SD. Statistical analysis was performed using one-way ANOVA followed by Tukey’s post hoc test, *n* = 6.

Figure 2a shows the results of the analysis of p53-positive cells in the injured cortical area following TBI in the control group and after administration of Na_2_S_2_O_3_ or aminooxyacetic acid (AOAA).

In the control group, the proportion of p53-positive cells in the ipsilateral cortex was approximately 33%, reflecting moderate p53 activation in response to traumatic injury. Administration of Na_2_S_2_O_3_ significantly reduced this value to around 22% (*p* < 0.05), indicating a potential protective effect of Na_2_S_2_O_3_ in attenuating p53 signaling activation. In contrast, in the AOAA-treated group, the proportion of p53-positive cells increased sharply to 50% (*p* < 0.05) (Figure 2b), suggesting enhanced p53 expression under inhibition of pyridoxal-dependent enzymes.

Analysis of nuclear p53 localization, which reflects its functional activity as a transcription factor, showed a similar trend. In the control group, about 24% of all cells exhibited nuclear p53 localization. Na_2_S_2_O_3_ administration nearly halved this figure to 13% (*p* < 0.05), while AOAA significantly increased nuclear localization to 52% (*p* < 0.05) (Figure 2c).

The proportion of p53-positive cells also exhibiting signs of nuclear fragmentation, characteristic of apoptosis, was evaluated. In the control group, this proportion was about 30%. After Na_2_S_2_O_3_ administration, it decreased to 20% (*p* < 0.05), whereas AOAA induced a significant increase to 45% (*p* < 0.05) (Figure 2d). These findings suggest that AOAA enhances the p53-mediated cell death pathway, while Na_2_S_2_O_3_ exerts a pronounced neuroprotective effect.

### 2.3. Assessment of the Level of Apoptosis

**Figure 3:**
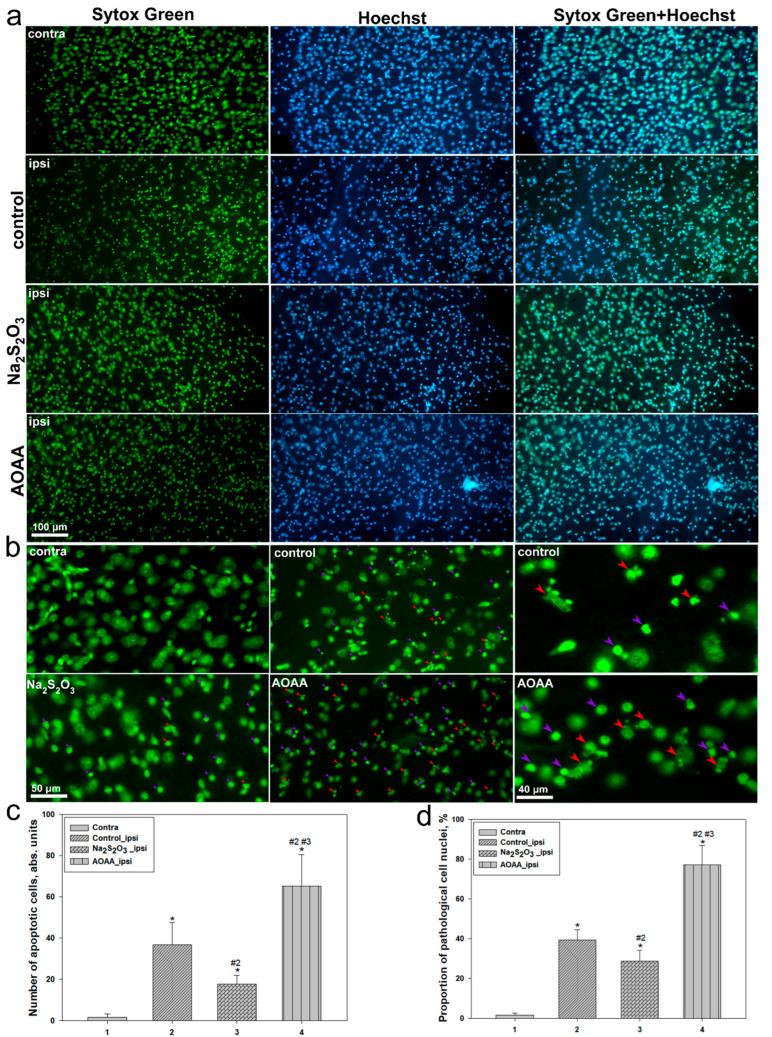
Assessment of nuclear pathology. (**a**) Fluorescence microscopy at ×200 magnification of the contralateral and ipsilateral hemispheres in the control group and following administration of Na_2_S_2_O_3_ or AOAA. Sections stained with Sytox Green (green) and Hoechst (blue). (**b**) Fluorescent images at ×400 magnification: Red arrows indicate nuclei with signs of fragmentation, while purple arrows mark nuclei without fragmentation but with pathological changes, such as chromatin condensation, reduced nucleoplasm volume, and altered nuclear shape. (**c**) Number of detected fragmented nuclei. (**d**) Quantitative evaluation of the proportion of pathologically altered cells in the contralateral and ipsilateral hemispheres across different groups. * *p* < 0.05—significant difference between ipsilateral and contralateral cortex; # *p* < 0.05—indicates a significant difference between the ipsilateral cortex of experimental and control groups. The numbers below the bars correspond to group labels (1: Contra; 2: Control_ipsi, 3: Na_2_S_2_O_3__ipsi, 4: AOAA_ipsi), while the significance markers (#2, #3) above the bars indicate statistically significant differences between the respective groups. Data are presented as mean ± SD. Statistical analysis was performed using one-way ANOVA followed by Tukey’s post hoc test, *n* = 6.

Analysis of the level of pathologically altered nuclei following TBI revealed significant differences between experimental groups, indicating the involvement of H_2_S in the regulation of apoptosis in the affected cortex (Figure 3a,b). In the contralateral hemisphere of the control group, cell morphology remained within normal limits. However, in the ipsilateral hemisphere of the control group, within the injury zone, the level of apoptosis increased significantly (*p* < 0.05) (Figure 3c), as evidenced by a marked rise in the number of cells with pyknotic and fragmented nuclei, characteristic of late-stage apoptosis (Figure 3a). This confirms the presence of pronounced neuronal damage in the injury area, associated with the activation of programmed cell death pathways.

Administration of Na_2_S_2_O_3_ resulted in a significant reduction in the number of apoptotic cells compared to the control group (*p* < 0.05) (Figure 3c). This group exhibited less nuclear fragmentation (Figure 3a) and a lower proportion of pathologically altered cells, suggesting a potential neuroprotective effect of Na_2_S_2_O_3_. However, the number of apoptotic nuclei remained higher than in the intact cortex.

Conversely, AOAA administration led to a sharp increase in the number of apoptotically altered cells (Figure 3a,c). AOAA caused the most pronounced rise in fragmented nuclei, accompanied by the highest percentage of pathologically altered cells among all groups (*p* < 0.05) (Figure 3c).

Similar trends were observed in the analysis of the proportion of pathologically altered cells. In the control group, this proportion was around 40%, significantly exceeding the values in the contralateral hemisphere (*p* < 0.05) (Figure 3d). Administration of Na_2_S_2_O_3_ reduced this percentage to approximately 20% (*p* < 0.05). In contrast, AOAA administration increased the proportion of pathologically altered cells to a maximum level of around 80% (Figure 3d).

### 2.4. Histological Examination

**Figure 4:**
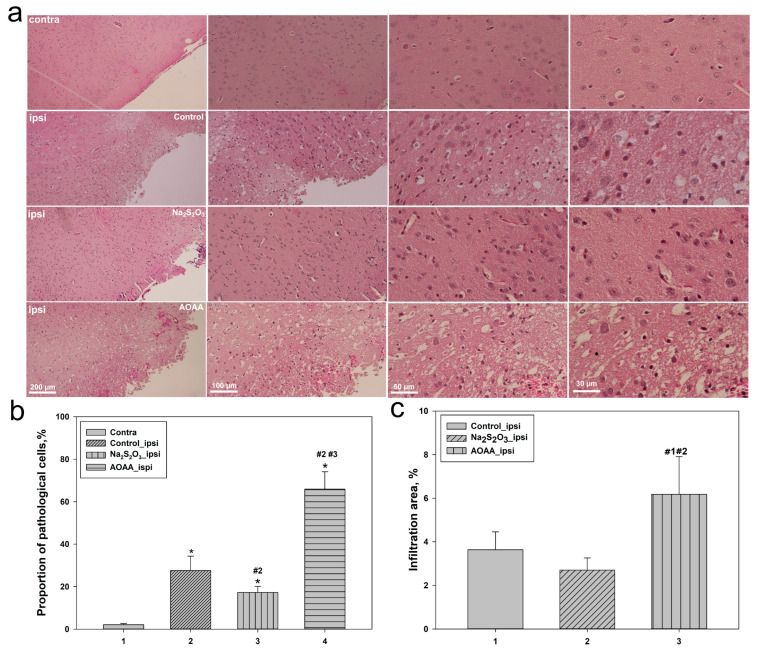
Histological assessment of brain damage in mice after TBI (hematoxylin and eosin staining). (**a**) Morphological changes in the contralateral and ipsilateral hemispheres in the control group and after administration of Na_2_S_2_O_3_ or AOAA. (**b**) Quantitative assessment of the proportion of pathologically altered cells in the contralateral and ipsilateral hemispheres across different groups. Pathologically altered cells were identified based on the presence of vacuolization, karyolysis, and pyknosis, which are characteristic of irreversible cellular damage. (**c**) Area of inflammatory infiltration in the injury zone in the control group and following Na_2_S_2_O_3_ or AOAA administration. “Infiltrated Area” refers to the measured area of inflammatory infiltration, identified based on the presence of dense clusters of leukocytes, as well as erythrocytes, indicating vascular damage. * *p* < 0.05—indicates a significant difference between the ipsilateral and contralateral cortex. # *p* < 0.05—indicates a significant difference between the ipsilateral cortex of experimental and control groups. The numbers below the bars correspond to group labels (1: Contra; 2: Control_ipsi, 3: Na_2_S_2_O_3__ipsi, 4: AOAA_ipsi), while the significance markers (#1, #2, #3) above the bars indicate statistically significant differences between the respective groups. Data are presented as mean ± SD. Statistical analysis was performed using one-way ANOVA followed by Tukey’s post hoc test, *n* = 6.

Histological analysis revealed pronounced morphological changes in brain tissue following TBI. In samples from the uninjured hemisphere, normal tissue architecture was preserved: no signs of degeneration, inflammation, or other pathological alterations were observed. In the control group, within the ipsilateral hemisphere at the injury site, significant damage was detected, including neuronal degeneration, cytoplasmic vacuolization, and inflammatory infiltration, indicating an active neuroinflammatory process (Figure 4a).

Administration of Na_2_S_2_O_3_ led to a noticeable reduction in the severity of tissue damage compared to the control group: neurons retained their morphology, and the number of vacuolated and pyknotic cells decreased, suggesting a possible neuroprotective effect. In contrast, administration of AOAA was associated with enhanced neurodegeneration, evident by cell fragmentation, an increased number of vacuolated neurons, and a substantial rise in inflammatory infiltration in the damaged area (Figure 4a).

Quantitative analysis supported these observations. The average proportion of pathologically altered cells in the contralateral hemisphere remained minimal across all groups, while in the injured zone of the control group, this value increased to approximately 30% (*p* < 0.05). Na_2_S_2_O_3_ administration reduced this to 20% (*p* < 0.05), confirming its protective effect. Conversely, in the AOAA group, the percentage of damaged cells rose sharply to 75% (*p* < 0.05) (Figure 4b), indicating exacerbated neurodegeneration upon CBS inhibition.

Analysis of the inflammatory response revealed similar trends. In the control group, the level of inflammatory cell infiltration was about 4%, which decreased to 2% with Na_2_S_2_O_3_ treatment, indicating its anti-inflammatory effect. The AOAA group exhibited the most pronounced inflammation, reaching approximately 8% (*p* < 0.05) (Figure 4c), confirming an intensified inflammatory response under conditions of reduced H_2_S levels.

### 2.5. Investigation of p53 Complexes with H_2_S Derivatives via Molecular Dynamics Simulations

**Figure 5:**
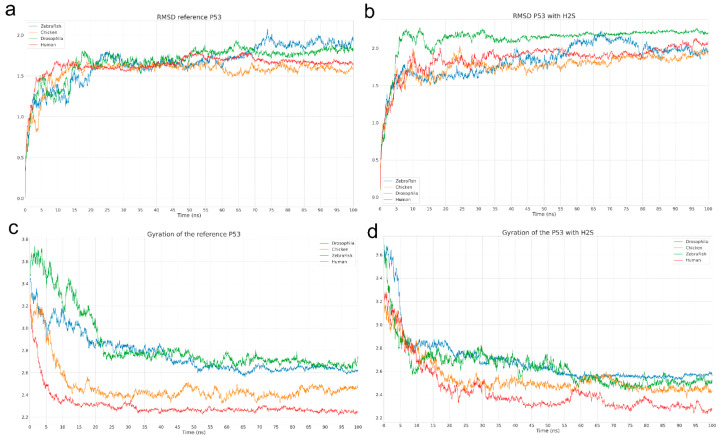
Analysis of H_2_S interaction with p53 using molecular dynamics. (**a**) RMSD plot for the reference system, (**b**) RMSD plot for the system with H_2_S, (**c**) Rg plot for the reference system, and (**d**) Rg plot for the system with H_2_S.

To assess the effect of hydrogen sulfide on the conformational properties of p53, molecular dynamics (MD) simulations were performed. A comparison of the root-mean-square deviation (RMSD) and radius of gyration (Rg) allowed us to determine the extent of structural changes in the protein in the reference system and in the presence of H_2_S (Figure 5).

Comparison of RMSD (Figure 5a,b) and Rg (Figure 5c,d) plots revealed no significant conformational differences either within or between the studied groups. In both cases, the proteins exhibited similar conformational evolution: after initial relaxation and equilibration, they reached a stable conformation within a relatively short time (~20 ns), as evidenced by the plateau in RMSD (Figure 5a,b).

Since the p53 structure in all four organisms contains a significant number of disordered polypeptide chains, RMSD values remain relatively high even for the equilibrium geometry. However, the core structural domains maintain their compactness and geometry in all cases.

The Rg plots (Figure 5c,d) show a general trend toward compaction of p53, likely due to the intrinsic dynamics of its domains. However, for human p53 in the presence of H_2_S, the Rg curve exhibits a wave-like oscillation instead of monotonic behavior, possibly indicating fluctuations between closely related conformations (Figure 5d).

Visual analysis of the MD trajectories did not reveal stable p53-H_2_S complex formation. However, the RMSD plot of human p53 (Figure 5b) displays periodic fluctuations, suggesting a long-range influence of H_2_S on protein dynamics, inducing oscillation between two conformational states.

We employed the MMPBSA method, which calculates the average interaction energy between the target protein and ligand over the entire simulation. The MMPBSA-derived binding energy indirectly accounts for energy barriers caused by ligand binding site recognition difficulties and the counteracting effects of Brownian motion of cytosolic ions at a given temperature. The MMPBSA analysis results are presented in Table 1 (Supplementary Materials).

In the first experiment, we placed H_2_S at a random location within the solvent box surrounding the p53 protein from one of the four species and conducted a simulation as described in the Materials and Methods section. As a result, we did not obtain energy values sufficient to suggest the formation of a stable p53–H_2_S complex. This aligns with our previous analysis, where no stable interactions between H_2_S and p53 were observed.

The second experiment was conducted only with human p53, as wave-like conformational fluctuations were observed in the RMSD plot in the presence of H_2_S. Given that the pKa of the H_2_S molecule is 7.05 and the average intracellular pH is approximately 7.4, it can be confidently stated that H_2_S predominantly exists in its dissociated form (anion) under physiological conditions, whereas under ischemic conditions, when the pH drops to ~6.5, it exists in its neutral form.

We performed molecular dynamics (MD) simulations of human p53 (Human) with H_2_S and HS^−^, as well as a protonated-at-histidine form (Human (prot)), which corresponds to its state at approximately pH 6.5.

The results of MM-PBSA and MM-GBSA calculations (Table 1) reveal significant differences in interaction energies between p53 and H_2_S versus HS^−^. In the Generalized Born (GB) model, the presence of neutral H_2_S leads to an energy decrease (−1.1648 kcal/mol), while HS^−^ has negligible impact (0.0784 kcal/mol). In the Poisson–Boltzmann (PB) model, the effect of H_2_S is again more pronounced (−0.3828 kcal/mol), in contrast to HS^−^ (0.1866 kcal/mol). The docking analysis for the protonated form of p53 (Human (prot)) did not reveal any significant stabilization effects.

**Figure 6:**
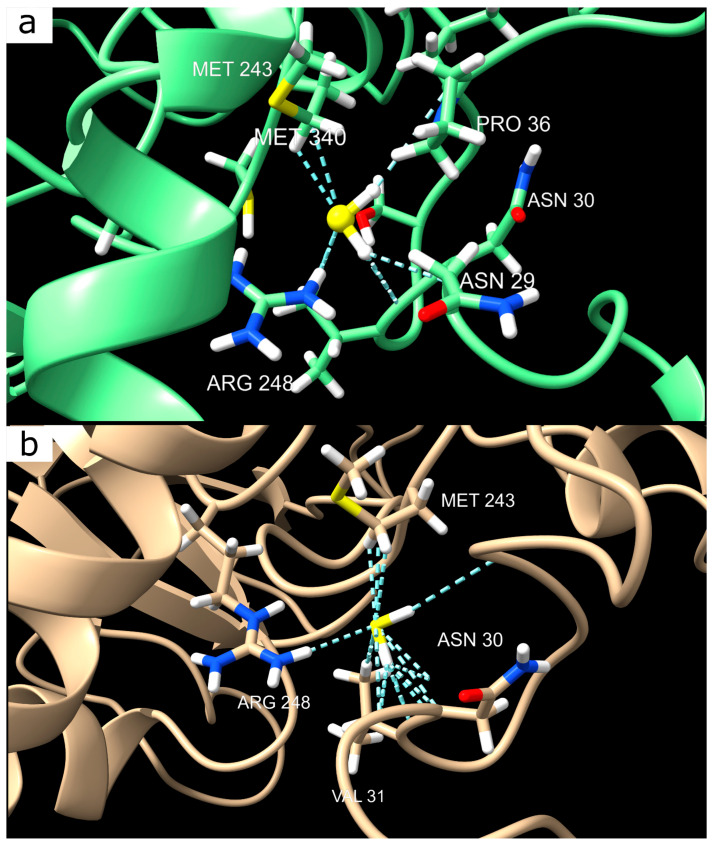
Predicted H_2_S-binding domains in the human p53 protein. (**a**) Putative H_2_S-binding domain in human p53. (**b**) Protonated form of the predicted H_2_S-binding domain in human p53. Cyan dashed lines represent van der Waals interactions, highlighting potential binding sites.

Despite the high variance, we tend to believe that van der Waals interactions between p53 and H_2_S are possible, even under protonation. The variance might be due to an unfavorable initial complex conformation or the influence of additional subtle intracellular conditions not captured in the simulations. H_2_S interacts with several key amino acids in p53. Among them are serine (SER 33), asparagine (ASN 30), proline (PRO 34), valine (VAL 31), arginine (ARG 248), and methionine (MET 243) (Figure 6a). These residues likely play a key role in stabilizing local structural changes occurring in p53 under the influence of H_2_S. Figure 6a clearly shows interactions between H_2_S and SER 33, as well as MET 243, which may be critical for complex formation and conformational modulation of p53. Docking analysis in the Human (prot) model also showed the most energetically favorable stabilization at the same site (Figure 6b).

The low stabilization energies observed in our MD simulations do not necessarily indicate complex instability but rather reflect the inherent limitations of the MD approach, which can struggle to capture subtle intermolecular interactions. More precise characterization of such interactions typically requires advanced quantum mechanics/molecular mechanics calculations, which are beyond the scope of the current study. Nevertheless, our docking results are promising: both the protonated and deprotonated forms of p53 exhibit binding to H_2_S within the same domain.

To assess the biological significance of this binding site, we conducted a literature analysis, which revealed that arginine 248 (Arg248) in human p53 is a clinically significant mutation hotspot [22]. Replacing this positively charged residue with a neutral glutamine (Gln248) leads to a loss of p53 function, increasing the likelihood of oncogenic mutations. This critical position is located within the DNA-binding domain of p53, directly affecting its ability to regulate gene expression and control cell survival.

H_2_S, known as a signaling molecule, has been identified as an inhibitor of p53 expression, contributing to neuronal survival in TBI by slowing the activation of p53-dependent apoptotic pathways [20]. Our findings suggest that H_2_S acts as a weak competitive inhibitor of p53, potentially interacting with its DNA-binding domain and thereby modulating its activity.

To test this hypothesis, we constructed a mutant form of protonated p53 (Human (mut)), in which Arg248 was substituted with Gln248. The results of the MM-PBSA analysis for this mutant (Table 1) revealed that this single amino acid substitution significantly disrupted the H_2_S binding preference, indicating that the Arg248 position is critically important for maintaining p53 activity.

The pronounced difference in stabilization energies of the p53 complex with the protonated and deprotonated forms of H_2_S may underlie an important signaling mechanism in neurons. At physiological pH, HS^−^ ions do not form complexes with p53 and thus do not affect its function. However, when pH drops below a critical threshold, the HS^−^ ion becomes protonated, allowing the neutral H_2_S molecule to initiate the formation of a multiprotein complex that may include not only p53 but also a DNA fragment or another transcription factor.

### 2.6. Bioinformatic Analysis of the Evolutionary Conservation of p53

We performed multiple sequence alignments of p53 amino acid residues in vertebrates and invertebrates to assess the conservation of this protein throughout the evolutionary development of species. In this study, the inclusion of evolutionary analysis was aimed at confirming the relevance of our molecular dynamics results, particularly regarding the potential of H_2_S to modulate p53 activity through interactions at highly conserved sites, such as Arg248. Understanding the conserved nature of these interactions is critical, as it supports the translational validity of our findings and underscores the broader biological significance of H_2_S-p53 interactions in pathological conditions, including TBI.

It was shown that the percentage of identity between *Homo sapiens* and *Bos taurus* reaches 81.56%, which is the highest value in the matrix. The similarity between *Mus musculus* and *Homo sapiens* is 78.04%, while between *Mus musculus* and *Bos taurus*, it is 75.00%. These high values highlight the close evolutionary relationship within the class Mammalia and indicate that the structure and function of the p53 protein in mammals are conserved due to shared genetic and physiological features (Table 2, Supplementary Materials).

Transitioning to fish, a moderate similarity between species can be observed. For example, the identity between *Danio rerio* and *Erpetoichthys calabaricus* reaches 60.11%, which is notably higher than their similarity to mammals, ranging between 47.84 and 51.86% (Table 2). This suggests a closer relationship within the Osteichthyes group and highlights the evolutionary divergence between fish and terrestrial vertebrates, likely due to differences in p53 adaptation to aquatic environments.

Among reptiles and birds, the results are also quite revealing. For instance, *Sphenodon punctatus* shows a similarity of 61.51% with *Gallus gallus* and 56.46% with *Catharus ustulatus*. Between birds themselves—chicken (*Gallus gallus*) and thrush (*Catharus ustulatus*)—the percentage of identity reaches 65.60% (Table 2), making this pair one of the closest in the matrix. These data confirm the evolutionary link between Aves and Reptilia, reflecting their common ancestry, and also indicate a high degree of p53 conservation within the bird class.

In insects, the similarity is noticeably lower but still stands out in cross-group comparisons. The identity between *Drosophila melanogaster* and *Bactrocera dorsalis* is 36.27%, which is significantly higher than their similarity to vertebrates (ranging from 19.94 to 24.15%, Table 2). This reflects the deep evolutionary divergence between arthropods and chordates while simultaneously highlighting a closer relationship within the Diptera order.

Comparisons between major organism groups reveal even more pronounced differences. The similarity between vertebrates and insects remains consistently low (19.94–24.15%), indicating a significant evolutionary split that occurred early in the development of multicellular animals. Among vertebrates, the lowest identity is observed between mammals and fish—for example, 47.84% between *Mus musculus* and *Erpetoichthys calabaricus* (Table 2). This corresponds to the earlier divergence of fish from the tetrapod lineage and reflects differences in p53 evolution between these groups.

**Figure 7:**
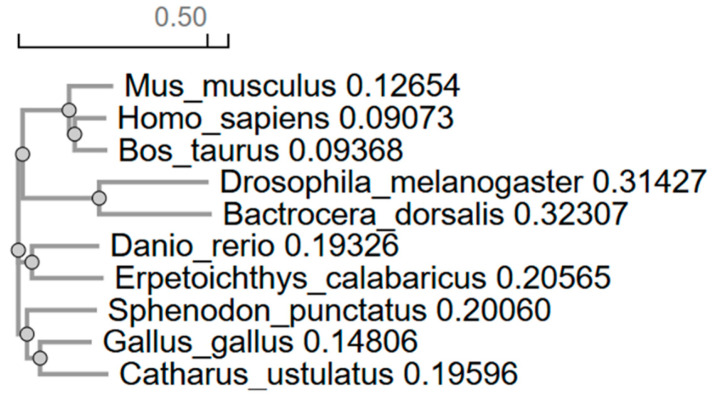
Phylogenetic tree calculated based on multiple comparisons for p53 from different organisms.

Additionally, to analyze evolutionary relationships between p53 in vertebrates and invertebrates, a phylogenetic tree was constructed based on multiple amino acid sequence alignments performed using the Clustal Omega method (Figure 7, Supplementary Materials).

The phylogenetic tree of the p53 protein reflects evolutionary relationships among different species. Mammals such as *Homo sapiens*, *Bos taurus*, and *Mus musculus* form a closely related cluster, where humans and cows show greater proximity to each other than to mice. This aligns with the expected evolutionary hierarchy among primates, even-toed ungulates, and rodents.

In contrast, insects like *Drosophila melanogaster* and *Bactrocera dorsalis* form a more distant cluster, with notable divergence between them, indicating significant evolutionary differentiation. Fish species, represented by *Danio rerio* and *Erpetoichthys calabaricus*, occupy an intermediate position, being closer to mammals than to insects, which is consistent with their place in vertebrate evolution.

Reptiles and birds, including *Sphenodon punctatus*, *Gallus gallus*, and *Catharus ustulatus*, also form a distinct group. Here, the tuatara appears more closely related to birds than to other clusters, while the birds themselves demonstrate a recent common ancestry.

### 2.7. Performance Evaluation of YOLO Variants in Nuclei Detection

Mean average precision at 50% IoU (mAP50) is the standard metric for evaluating object detection performance. The results, as provided in Table 3, indicate that YOLOv8 achieved a mAP50 of 0.727, significantly higher than YOLOv11′s 0.615, suggesting superior localization accuracy. This is particularly relevant for biomedical applications where precise cell detection is essential. The mAP50-95 score for YOLOv8 (0.177) also surpassed YOLOv11 (0.119). However, in cases of class imbalance, where normal nuclei significantly outnumber pathological nuclei, the F1-score provides a more reliable measure. Notably, YOLOv11 achieves a high sensitivity of 82.2% (recall > 80%) and an F1-score of 62.7%, indicating that most target objects are correctly identified in the predicted outputs.

Table 4 presents a comparison of preprocessing methods and hyperparameters for YOLOv8 and YOLOv11. For YOLOv8, the highest F1-score (45.1%) was achieved at epoch 225 using mosaic images and standard YOLO augmentations, without grayscale or class weights, whereas introducing weights [4.0, 1.0] reduced performance to 38.6% (epoch 200). For YOLOv11, the peak F1-score (62.7%) occurred at epoch 350 with grayscale images, Roboflow augmentations, mosaic, and standard YOLO augmentations, but no class weights, while the lowest score (34.1%) was observed with weights and mosaics at epoch 208. Transitioning to grayscale markedly improved YOLOv11 performance (62.7% versus 50.5% without grayscale). Class weights [4.0, 1.0] consistently diminished F1-scores across both models (e.g., from 50.5% to 34.1% for YOLOv11). Disabling mosaic and YOLO augmentations reduced performance (34.5% for YOLOv11), whereas reinstating them enhanced metrics.

**Figure 8:**
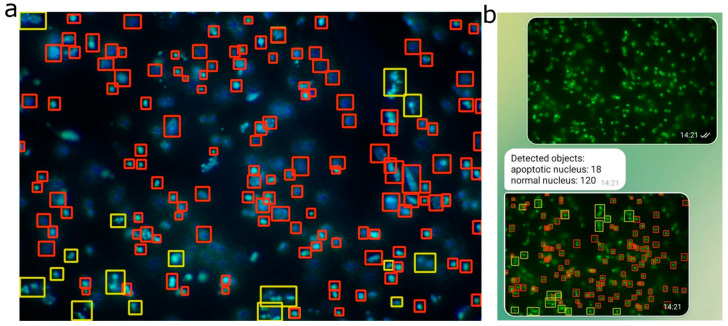
Detection of normal and apoptotic nuclei using a YOLO model on fluorescence microscopy images. (**a**) A microscopy field stained with Hoechst dye (blue) visualizes cell nuclei. Normal nuclei are marked with red bounding boxes, while apoptotic nuclei are indicated by yellow boxes. (**b**) Demonstration of a Telegram bot for automated nuclear counting. The top image shows a fluorescence field with nuclei stained by Sytox Green (green). The bottom panel overlays YOLO detection results: 18 apoptotic nuclei (yellow boxes) and 120 normal nuclei (red boxes).

Deployment via Telegram bots showcased YOLOv11′s real-time processing strengths, aligning with its reputation for rapid image handling. This practical integration underscored the model’s utility in high-throughput, user-facing biological analysis systems. To illustrate the practical application of the trained YOLO model in the analysis of fragmented nuclei, Figure 8 presents examples of nuclear detection in fluorescence microscopy images, as well as its integration into a user-friendly interface.

Based on the obtained data, our study confirms the hypothesis that H_2_S-dependent signaling pathways significantly influence apoptotic cell death in nervous tissue following TBI, possibly through cytoplasmic–nuclear regulation of p53 expression. The main findings include the following:

1. Following TBI, p53 expression significantly increased in the ipsilateral cortex, showing cell type-specific localization: predominantly cytoplasmic in neurons, indicating activation of the mitochondrial pathway, and both cytoplasmic and nuclear in astrocytes, suggesting transcription-dependent apoptotic signaling.
2. Sytox Green staining confirmed that cytoplasmic p53 accumulation in neural cells is associated with early apoptotic markers, while nuclear p53 correlates with more advanced stages of apoptosis. Quantitative analysis revealed a marked decrease in the number of p53-positive cells and apoptotic features following Na_2_S_2_O_3_ administration, highlighting its neuroprotective effect. In contrast, cystathionine-β-synthase (CBS) inhibition by AOAA sharply increased p53 nuclear localization and apoptosis, indicating the critical role of H_2_S in suppressing p53-driven cell death.
3. Sequence analysis of p53 across different species confirmed high conservation in vertebrates, underscoring the fundamental role of this protein in regulating cell fate, while significant divergence in invertebrates reflects their distinct apoptotic mechanisms.
4. Molecular dynamics simulations showed that, while stable H_2_S-p53 complexes do not form, weak, transient interactions involving critical residues such as Arg248 may modulate the conformational flexibility of p53, particularly under conditions of reduced pH. This supports a model in which H_2_S acts as a fine-tuner of p53 activity under TBI conditions.
5. The development of a YOLOv11-based neural network significantly improved the accuracy and speed of apoptotic cell recognition in fluorescence microscopy images, providing a scalable approach for quantitative morphometric analysis.

## 3. Discussion

TBI is accompanied by a cascade of molecular and cellular disturbances, including the activation of programmed cell death, inflammation, and disruption of neural network integrity. One of the key regulators of the cellular response to injury is the transcription factor p53, which plays a critical role in the control of apoptosis. At the same time, the signaling molecule H_2_S is now recognized as an important neuromodulator with pronounced neuroprotective properties. Investigating the interplay between H_2_S and p53 is of particular interest in the context of TBI, as their interaction may critically influence neuronal survival and brain tissue recovery [23].

The data obtained in our study demonstrate a close relationship between the intracellular localization of p53 and morphological signs of neural tissue damage after TBI. Three-dimensional visualization of p53 distribution using confocal laser scanning microscopy revealed fundamental differences between the intact and injured hemispheres. In the intact cortex, p53 was detected at extremely low levels. It is known that p53 expression under normal conditions is tightly regulated by proteasomal degradation mechanisms, primarily through ubiquitination mediated by the E3 ubiquitin ligase MDM2, which promotes rapid cytoplasmic clearance of p53 and maintains its basal levels [20,24]. In addition to ubiquitination, p53 stability and activity are further modulated by a variety of post-translational modifications, including phosphorylation, acetylation [25], and methylation [26]. These modifications alter the protein’s conformation, DNA-binding capacity, and interactions with coactivators and inhibitors, as well as its subcellular localization. Moreover, the functional status of p53 is influenced by interactions with various proteins, including transcription regulators, chaperones, and DNA repair factors. However, under cellular stress conditions, including traumatic impact, signaling cascades are triggered that inhibit MDM2 activity, stabilize the p53 molecule, and promote its rapid activation. As a result, p53 rapidly accumulates in the cell, functioning as a transcription factor that initiates processes such as apoptosis [27,28,29].

Following TBI, there was a marked increase in p53 expression in the ipsilateral cortex, with its localization showing distinct cell-type-specific patterns: in neurons, p53 predominantly accumulated in the cytoplasm, whereas in astrocytes, it was present in both the cytoplasm and the nucleus. These differences may indicate the presence of alternative regulatory mechanisms of p53 in neurons and glia. Cytoplasmic accumulation of p53 in neurons is likely related to its role in activating the mitochondrial-dependent apoptotic pathway. Under stress, activated p53 can translocate to the mitochondria, where it facilitates oligomerization of BCL-2-Associated X Protein (Bax) and BCL-2 Antagonist/Killer (Bak), increasing mitochondrial membrane permeability and promoting cytochrome c release, thereby initiating apoptosis [30]. In contrast, nuclear localization of p53 in astrocytes may reflect the activation of the classical apoptotic cascade, triggered by transcription-dependent p53 mechanisms. Under stress, p53 induces the expression of pro-apoptotic proteins such as Bax, p53 Upregulated Modulator of Apoptosis (PUMA), and Phorbol-12-Myristate-13-Acetate-Induced Protein 1 (Noxa), which compromise mitochondrial membrane integrity and lead to apoptotic cell death [20,31].

Staining of cell nuclei with the Sytox Green marker and subsequent 3D reconstruction of cell morphology enabled the correlation of p53 localization with different stages of cell death. Neurons showing predominantly cytoplasmic p53 expression more often exhibited early signs of apoptosis, such as partial chromatin condensation and mild nuclear shrinkage [32]. In contrast, astrocytes with strong nuclear p53 expression displayed more advanced stages of apoptosis: nuclear fragmentation, formation of apoptotic bodies, and membrane changes [33], possibly reflecting a more active role of p53 in initiating transcriptionally mediated apoptosis in these cells.

These observations are further supported by quantitative analysis from fluorescence microscopy. In the control group, p53-positive cells accounted for approximately 33% of all Hoechst-stained nuclei, with only 24% of these showing nuclear localization. Around 30% of p53-positive cells exhibited signs of nuclear fragmentation, indicating a moderate level of p53-driven apoptosis. Administration of Na_2_S_2_O_3_ significantly altered this profile: p53 expression decreased to 22%, and the proportion of cells with nuclear localization nearly halved. The number of p53-positive cells exhibiting apoptotic features also significantly decreased, indicating a protective effect of Na_2_S_2_O_3_, likely mediated through restoration of H_2_S levels and inhibition of p53-dependent apoptosis [34].

The opposite effect was observed following the administration of AOAA, an inhibitor of the CBS enzyme responsible for endogenous H_2_S synthesis [35]. In this group, the proportion of p53-positive cells sharply increased to 50%, with more than half of them displaying nuclear localization of the protein, and 45% showing signs of nuclear fragmentation. This may indicate that a decrease in H_2_S levels promotes enhanced activation of p53 and triggers cell death cascades in both neurons and astrocytes. Under H_2_S-deficient conditions, redox homeostasis may be disrupted, mitochondrial stress may intensify [3,36], and p53 may become activated as a central regulator of the cellular damage response [37].

Further confirmation of these conclusions was provided by morphological analysis of nuclei and histological examination of brain tissue. In the lesion area of the control group, the level of apoptosis significantly increased compared to the contralateral side, corresponding to a rise in the number of cells with fragmented nuclei and altered nuclear morphology. Na_2_S_2_O_3_ significantly reduced these indicators, whereas AOAA exacerbated apoptotic manifestations, increasing the proportion of pathologically altered nuclei to 80%. A similar trend was observed in the overall assessment of brain tissue damage: the AOAA group exhibited the most severe neurodegenerative changes and pronounced inflammation, while Na_2_S_2_O_3_ showed clear neuroprotective and anti-inflammatory potential [38].

Taken together, the results of three-dimensional and quantitative analysis of p53 expression, nuclear morphology, and brain tissue histology demonstrate that p53 activation following TBI is associated with the development of apoptotic processes in the injured cortex. Increased nuclear expression of p53 and its correlation with signs of nuclear fragmentation indicate the involvement of this protein in cell death, whereas Na_2_S_2_O_3_ mitigates these effects, providing a protective benefit. In contrast, inhibition of H_2_S biosynthesis with AOAA leads to enhanced p53 expression and apoptosis, highlighting the crucial role of H_2_S in regulating p53-dependent stress responses in the brain following injury.

The molecular dynamics analysis enabled evaluation of the impact of H_2_S on the conformational properties of the p53 protein. Comparison of RMSD and Rg between the reference system and the system with H_2_S revealed no significant structural differences, indicating the overall stability of p53 is preserved in the presence of H_2_S. However, a key observation was a change in the Rg curve pattern for human p53 upon H_2_S addition: instead of a monotonic trend, wave-like oscillations were observed, potentially reflecting transitions between closely related conformational states. This is consistent with the RMSD data, where periodic fluctuations were also noted for human p53 in the presence of H_2_S, likely indicating dynamic domain rearrangements without full structural destabilization.

Interestingly, visual analysis did not confirm the formation of stable complexes between H_2_S and p53, which is further supported by MM-PBSA calculations. However, our docking results revealed specific, albeit weak, van der Waals interactions involving key residues, including serine (Ser33), asparagine (Asn30), proline (Pro34), valine (Val31), arginine (Arg248), and methionine (Met243). These residues likely play a critical role in local structural stabilization under H_2_S influence. Notably, Arg248, a known mutation hotspot in p53, was identified as a significant contributor to this interaction, and its substitution with neutral glutamine (Gln248) significantly disrupted the H_2_S binding preference. Given the functional importance of Arg248 in DNA binding, this substitution could substantially impact the neuroprotective role of p53 following TBI, potentially reducing its ability to regulate the transcription of genes involved in cell survival and apoptosis.

Interestingly, visual analysis did not confirm the formation of stable complexes between H_2_S and p53, which is further supported by MMPBSA calculations. In the first experiment, where H_2_S was randomly positioned near p53, the interaction energy was insufficient to stabilize a complex, regardless of the organism. This suggests that H_2_S does not bind p53 via classical specific sites. However, the second experiment, focused on human p53, revealed differences in the behavior of neutral and anionic forms of H_2_S. In the GB model, the neutral H_2_S molecule showed a weak but statistically significant reduction in interaction energy (−1.1648 kcal/mol), whereas HS^−^ had virtually no effect. A similar trend was observed in the PB model, where H_2_S again demonstrated greater activity (−0.3828 kcal/mol versus 0.1866 kcal/mol for HS^−^).

This observation aligns with the known physiological pH-dependence of H_2_S speciation. At normal physiological pH (~7.4), H_2_S predominantly exists in the form of HS^−^, which, as our calculations indicate, does not significantly interact with p53. However, under ischemic conditions, where the pH can drop to ~6.5, the proportion of neutral H_2_S increases, potentially facilitating transient interactions with p53, particularly through the Arg248 site. This shift may represent a key signaling mechanism, allowing H_2_S to modulate p53 activity under stress conditions, such as TBI or other pathological states characterized by acidosis.

These results are consistent with physiological conditions: at normal pH, H_2_S predominantly exists in the form of HS^−^, which, according to the data, does not interact with p53 [23,37]. However, under ischemic conditions, where the pH drops to around 6.5 [39], the proportion of the neutral H_2_S form increases, which, as calculations show, can modulate the conformational dynamics of the protein. This may explain a potential signaling mechanism in which H_2_S acts as a metabolic sensor, indirectly influencing p53 function through changes in its structural flexibility. The observed oscillations in RMSD and Rg, as well as weak van der Waals interactions, suggest that H_2_S likely does not bind to isolated p53, but may participate in the formation of multicomponent complexes involving DNA or other transcription factors, especially under oxidative stress conditions.

It is worth noting that this subtle modulation of p53 by H_2_S could have significant biological implications. Given that Arg248 is a critical residue within the DNA-binding domain of p53, even transient interactions with H_2_S may influence the transcriptional activity of this protein. This is particularly relevant in the context of neuronal survival, where p53 plays a central role in apoptotic signaling pathways. The competitive binding of H_2_S to Arg248 may reduce p53′s DNA affinity, thereby modulating its pro-apoptotic functions, potentially contributing to neuroprotection following TBI.

It is worth noting that the high variance in the MMPBSA data, particularly for H_2_S, may be related to the method’s limitations, such as dependence on the initial conformation or the neglect of solvent dynamics and ionic effects in vivo [40]. Nevertheless, the identified trends support the hypothesis of pH-dependent effects of H_2_S on p53. The data highlight that even weak interactions, which do not result in stable complex formation, may still regulate protein function by altering its dynamic properties. This underscores the role of H_2_S in the fine-tuning of cellular processes, particularly under stress conditions where acidity and redox balance undergo significant changes.

Modulation of p53 structure by H_2_S may influence its transcriptional activity and its involvement in regulating cellular processes such as apoptosis, DNA repair, and more. Even weak van der Waals interactions can significantly affect conformational changes in p53 [11,12], which in turn may represent one of the key mechanisms in H_2_S-mediated neuroprotection following TBI.

Additionally, analysis of the evolutionary conservation of the p53 protein among vertebrates and invertebrates revealed a clear correlation between the degree of amino acid sequence similarity and the evolutionary relatedness of species. The highest identity was observed within the mammalian class: 81.56% between *Homo sapiens* and *Bos taurus*, and 78.04% between humans and *Mus musculus*, underscoring their close phylogenetic relationship. These high values are consistent with the conservation of p53 structure and function, which are critical for the regulation of the cell cycle, DNA repair, and apoptosis [11,27]. It is likely that natural selection has preserved key domains of the protein, preventing mutations that could disrupt its interactions with partner molecules such as MDM2 or DNA binding sites [41].

Interestingly, *Bos taurus* shows slightly closer similarity to primates than to rodents, which may reflect differences in evolutionary rates or adaptations related to body size and metabolism. Among fish species, the identity between *Danio rerio* and *Erpetoichthys calabaricus* (60.11%) significantly exceeds their similarity to mammals (47–52%), confirming the distinct evolutionary path of bony fish and their early divergence from tetrapod ancestors. The moderate conservation of p53 in this group is likely due to adaptations to aquatic environments, where p53 may have acquired specialized functions. At the same time, the greater similarity between fish and mammals than between fish and insects emphasizes their shared vertebrate ancestry, despite the deep divergence between these groups [42,43].

Reptiles and birds exhibit the expected evolutionary continuity. The high identity values between *Sphenodon punctatus* and birds (61.51% with *Gallus gallus*), as well as between the bird species themselves (65.60% between *Gallus gallus* and *Catharus ustulatus*), support the hypothesis that Aves originated from reptiles. The conservation of p53 in this class likely reflects shared mechanisms of metabolic regulation, essential for sustaining the high activity levels typical of birds. Interestingly, *Sphenodon punctatus*, despite its archaic features, is genetically closer to birds than to other reptiles, consistent with data on the slow pace of molecular evolution in this species [44].

Insects, represented by *Drosophila melanogaster* and *Bactrocera dorsalis*, form a distinct branch with minimal similarity to vertebrates (19.94–24.15%), corresponding to their early evolutionary divergence. However, within the dipteran group, identity reaches 36.27%, indicating the preservation of fundamental p53 functions, such as maintaining genomic stability, even amidst significant structural divergence. The low similarity values between arthropods and chordates may be linked to differences in apoptotic mechanisms or adaptation to other forms of cellular stress [45].

The phylogenetic tree constructed from multiple sequence alignments fully aligns with the identity matrix, confirming the reliability of the results. The clustering of mammals, birds, fish, and insects into separate nodes reflects commonly accepted taxonomy, while the intermediate position of reptiles between fish and birds highlights their role in the evolutionary transition to terrestrial vertebrates.

These findings emphasize that p53 conservation correlates with evolutionary distance between species—preserving key functional domains while allowing variation in less critical regions. Differences between groups may reflect adaptation to specific ecological niches, such as aquatic environments in fish or flight in birds. The low similarity with insects points to a deep divergence in molecular mechanisms; however, the retention of a p53 homolog even in such distantly related groups confirms its fundamental role in maintaining cellular homeostasis.

Additionally, within the framework of this study, a neural network model based on YOLOv11 was developed for automated recognition and counting of fragmented nuclei in images obtained via fluorescence microscopy. This significantly accelerated morphological profiling of apoptosis and enabled quantitative assessment with high reproducibility. Nuclear fragmentation is a morphological marker of the late stages of apoptosis, commonly observed in conditions of neurodegeneration [33]. Assessing the degree of nuclear fragmentation provides crucial insights into the extent of cell death and the effectiveness of therapeutic interventions. However, traditional methods of analyzing fluorescent images are time-consuming and prone to subjective interpretation.

Our results show that the optimal performance of the model depends on the specific combination of preprocessing and hyperparameter tuning. YOLO 11 outperformed YOLO 8 (F1-score of 62.7% versus 45.1%), potentially reflecting architectural advancements. The shift to grayscale proved particularly effective for YOLO 11, likely by simplifying the color space, reducing noise, and enhancing object detection in biological images. The annotation process, involving maximized contrast and shadows with adjusted brightness, ensured object visibility, while assigning visually distinct images to the validation set likely improved model robustness. Contrary to expectations, weighting the underrepresented apoptosis class diminished F1-scores, suggesting possible overfitting to this class and a subsequent loss of generalization. Removing weights and restoring mosaic and augmentations restored class balance and improved detection quality. Disabling mosaic and YOLO augmentations during preprocessing lowered F1-scores, highlighting their role in generating diverse training samples. The best outcomes emerged from integrating grayscale, additional augmentations, and mosaics, affirming the value of a multifaceted data-processing strategy. Future investigations should explore alternative augmentation techniques and hyperparameter optimizations, such as adjusting learning rates or incorporating regularization, to further elevate YOLO performance on biological imagery.

The moderate F1-scores observed across all models may be partly attributed to the occurrence of false-positive detections, which are reflected in the precision metric. High fluorescence intensity from the underlying nuclei can increase the likelihood of background noise being incorrectly classified as positive signals. In the context of nuclear detection—particularly for apoptotic nuclei in biomedical research—high sensitivity is often prioritized over maximal average precision (all mAP metrics). This is because identifying as many true nuclei as possible is crucial, especially when downstream validation steps can compensate for false positives. In this study, YOLOv11 demonstrated a sensitivity of 82.0%, significantly higher than all other evaluated models. This high sensitivity implies a reduced likelihood of missing apoptotic nuclei, aligning well with the primary objectives of most diagnostic and research workflows focused on apoptosis. F1-scores, in particular, are widely regarded as a reliable performance metric in biomedical imaging tasks involving nuclear detection, as noted in the prior literature [46,47,48,49].

These results affirm YOLOv11′s suitability for morphological profiling in fluorescence microscopy, building on its established efficacy in medical imaging benchmarks [19]; however, on fluorescent cell images, it has not shown the expected result. The modest mAP@0.5–0.95 reveals persistent difficulties in exact localization, particularly in crowded areas.

Early val/cls_loss instability underscores the need for meticulous data curation in microscopy pipelines. The expanded augmentation suite curbed overfitting effectively, though spatial noise and photobleaching likely fueled transient learning volatility. The small dataset size (37 images, ~3200 labeled nuclei) is a notable limitation, potentially amplifying early instability and constraining mAP@0.5–0.95 performance. Overall, this study suggests that YOLOv11, despite a lower validation mAP, demonstrates superior performance in terms of the F1-score on the validation set, as well as in subjective evaluations of predicted objects during inference under consistent hyperparameters. The high sensitivity of YOLOv11 indicates its potential for broader applicability, including the analysis of fluorescent nuclei in other disease models, such as cancer or neurodegenerative disorders, provided that sufficiently diverse training datasets are employed. Future work could leverage transfer learning from larger microscopy datasets or synthetic data generation to enhance robustness. The single-annotator approach, while consistent, risks subtle biases in interpreting “bubble” features; inter-annotator validation or automated pre-filtering (e.g., edge detection) could refine label accuracy. The dropout-free design preserved critical nuclear boundary details, vital for separating apoptotic bodies from background fragments. Telegram’s role as a deployment platform further highlighted YOLO’s architectural efficiency, minimizing latency while maximizing accessibility—a boon for real-time biological research tools.

## 4. Materials and Methods

### 4.1. Animals and Ethical Approval

The experiments focused on studying traumatic brain injury were conducted on adult male CD-1 mice, aged 14–15 weeks, with a body weight ranging from 20 to 25 g. The animals were housed in comfortable conditions, in groups of 6–7 individuals in spacious cages, with unlimited access to food and water. The facility maintained a stable ambient temperature of 22–25 °C, and ventilation provided 18 complete air changes per hour, ensuring an optimal environment for animal welfare.

All procedures were carried out in accordance with strict ethical standards, as defined by international, national, and institutional regulations on the use of laboratory animals. The study complied with the provisions of Council Directive 86/609/EEC of 24 November 1986 on the protection of animals used for experimental and other scientific purposes, as well as with Russian regulations, including the “Rules of Laboratory Practice” (Order No. 708n of the Ministry of Health of the Russian Federation dated 23 August 2010) and GOST 33215–2014, which defines requirements for facility equipment and the organization of procedures involving animals.

The study was approved by Protocol No. 2 of the Bioethics Committee of Don State Technical University, dated 17 February 2020, confirming its compliance with high ethical and scientific standards.

### 4.2. Objects and Procedure

To model severe TBI, a custom-developed injury model was employed. Anesthesia was administered using the following protocol: intramuscular injection of a mixture of Xyla (0.2 mL/kg, 2% xylazine hydrochloride solution; Interchemie Werken “de Adelaar” BV, Venray, The Netherlands) and Zoletil (15 mg/kg, a combination of tiletamine hydrochloride and zolazepam hydrochloride; Virbac, Carros, France). Anesthesia was verified by the absence of a pain response (paw pinch) and the loss of corneal reflex.

**Figure 9:**
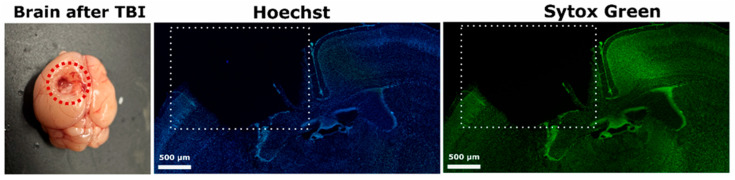
Modeling of TBI with extensive damage. The image shows a mouse brain with the trauma-affected area highlighted, along with histological sections stained with Hoechst and Sytox Green. The red dashed circle indicates the region of direct mechanical impact, while white dashed rectangles highlight the injury zone visible on histological sections.

The fur on the head was shaved, and the skin was thoroughly disinfected to prepare for surgery. Mice were placed in a special apparatus designed for injury modeling. A midline incision was made to expose the skull. The head was fixed to ensure accurate targeting, and a 3 mm diameter hole was drilled using a microdrill. A metal rod (2 mm tip diameter, 3 mm length, and 150 mg weight) was dropped from a height of 1 cm into the hole. The drop coordinates were defined as 2 mm dorsal to bregma and 1 mm lateral to the midline, targeting the parietal cortex (Figure 9). The site was then rinsed with sterile saline and sealed with bone wax. The wound was sutured.

To investigate the role of H_2_S in regulating p53 protein after TBI, the following agents were used: the H_2_S donor—sodium thiosulfate (1000 mg/kg Na_2_S_2_O_3_; Moskhimpharmpreparaty, named after N.A. Semashko) [50], and the cystathionine-β-synthase (CBS) inhibitor—aminooxyacetic acid (AOAA, 5 mg/kg; Tianjin Xidian Chemical Technology Co., Ltd., Tianjin, China) [35]. These compounds were administered intraperitoneally after TBI, and then daily until decapitation. The control group received physiological saline.

### 4.3. Laser Scanning Confocal Microscopy

To study the localization of p53 protein in brain tissue 24 h after TBI, the following methodological approach was applied. Animals were anesthetized, followed by perfusion through the right ventricle of the heart with a 4% paraformaldehyde (PFA) solution. The mice were then positioned head-down for 2 h to ensure even distribution of the fixative. After this, the brain was extracted and post-fixed in 4% PFA for an additional 12 h to complete tissue fixation. A frontal brain section 0.4 cm thick, including the necrotic zone resulting from TBI, was prepared. Thin slices, approximately 20 μm thick, were obtained using a Leica VT 1000 S vibratome (Leica Biosystems, Nussloch, Germany). The slices were sequentially incubated in 15% and 30% sucrose solutions for 1 h each for cryoprotection and then frozen at −80 °C.

After washing in phosphate-buffered saline (PBS), the sections were incubated in a blocking solution (5% bovine serum albumin (BSA, Sisco Research Laboratories Pvt. Ltd., Mumbai, India) and 0.3% Triton X-100 (Sisco Research Laboratories Pvt. Ltd., Mumbai, India)) for 1 h at room temperature to reduce nonspecific antibody binding. This was followed by incubation with primary antibodies: rabbit anti-p53 antibodies (1:100, PAA928Mu01, Cloud-Clone Corp, Wuhan, China), and either mouse anti-NeuN (neuronal nuclear protein; 1:1000; FNab10266, FineTest, Wuhan, China) or mouse anti-GFAP (astrocyte marker; 1:1000, SAB4200571, Sigma-Aldrich, St. Louis, MO, USA) antibodies for 48 h at 4 °C. Following multiple washes in PBS, the slices were incubated with secondary antibodies: anti-rabbit IgG (H + L) Abberior STAR 635P (1:500, Abberior GmbH, Göttingen, Germany) and anti-mouse IgG (H + L) Abberior STAR 580 (1:500, Abberior GmbH, Göttingen, Germany). Negative controls included sections processed without primary antibodies.

To stain nuclei of neurons and glial cells, Sytox Green Stain (ThermoFisher Scientific, Waltham, MA, USA) was used at a working concentration of 1:1000 in PBS. Sections were incubated in this solution for 20–30 min at room temperature in the dark, allowing for specific staining of nuclei with compromised membranes. After staining, the samples were washed three times in PBS to remove excess dye and reduce background fluorescence. They were then mounted in an antifade medium (Abberior GmbH, Göttingen, Germany) to prevent photobleaching of fluorescent signals during prolonged imaging. Finally, coverslips were applied to protect the sections from drying and to optimize imaging conditions.

Analysis was performed using an inverted confocal laser scanning microscope (Abberior Facility Line, Abberior Instruments GmbH, Germany), providing high spatial resolution for examining cellular structures. For 3D reconstruction, Z-scanning was conducted with a step size of 200 nm and a pixel size of 40 nm. Three-dimensional image reconstruction was carried out using ImageJ software (version 1.54j, National Institutes of Health, Bethesda, MD, USA).

### 4.4. Double Staining for Assessing Apoptosis

In this study, we analyzed morphological changes in the nuclei of neurons and glial cells under conditions of TBI. The primary objective was to determine the extent of cellular damage and the severity of apoptosis across different experimental groups. Two fluorescent dyes—Hoechst 33342 (Sisco Research Laboratories Pvt. Ltd., Mumbai, India) and Sytox Green (ThermoFisher Scientific, Waltham, MA, USA)—were used for nuclear visualization.

Hoechst 33342 has a unique ability to permeate intact cell membranes, making it suitable for both live and fixed cells. It specifically binds to AT-rich regions in the minor groove of DNA without requiring prior denaturation, providing high selectivity for nuclear staining. When excited by ultraviolet light at approximately 350 nm, it emits a bright blue fluorescence with a maximum emission at 461 nm, making it ideal for fluorescence microscopy. The intensity of the fluorescence is proportional to DNA content, allowing Hoechst 33342 to be used not only for visualizing nuclear structures, but also for cell cycle analysis or apoptosis detection [51].

In contrast, Sytox Green is a membrane-impermeant nucleic acid stain that only penetrates cells with compromised plasma membrane integrity. It does not enter intact cells; thus, its accumulation in the nucleus indicates late-stage apoptosis or necrosis. When excited with green light at 488 nm, Sytox Green emits an intense green fluorescence with a peak emission around 523 nm. In this protocol, Sytox Green served as an additional nuclear marker, enhancing the accuracy of identifying nuclei with pronounced pathological changes [52].

Brain samples were pre-fixed in 4% PFA overnight at 4 °C to preserve the morphological structure of the tissue. After fixation, slices approximately 20 μm thick were prepared using a Leica VT 1000 S vibratome (Leica Biosystems, Nussloch, Germany). The slices were then stained with Hoechst 33342 (Sisco Research Laboratories Pvt. Ltd., Mumbai, India) at a concentration of 40 μM and Sytox Green (ThermoFisher Scientific, Waltham, MA, USA) at 1 μM for 10 min.

The stained sections were mounted in a 60% glycerol solution, which served as a clearing and preservation medium, improving the quality of the fluorescent signal. The analysis was conducted using fluorescence microscopes Olympus BX53 (Olympus Corporation, Tokyo, Japan) and Altami LUM 1 (Ningbo Haishu Honyu Opto-Electro Co., Ltd., Ningbo, China, in collaboration with Altami Ltd., St. Petersburg, Russia). The microscopes were equipped with a high-resolution digital camera (EXCCD01400KPA, Hangzhou ToupTek Photonics Co., Ltd., Hangzhou, China), which enabled the acquisition of detailed images of nuclear staining with high resolution and contrast.

For quantitative analysis of nuclear abnormalities, each image at ×200 magnification was assessed by counting the absolute number of fragmented nuclei, as well as calculating the proportion of pathologically altered nuclei, expressed as the percentage of nuclei showing pathological changes relative to the total number of stained nuclei in the image, using the following Formula (1):

(1) $$P_{path}=\frac{N_{path}}{N_{total}}\times 100$$

In this formula, N*_path_* represents the number of nuclei exhibiting signs of fragmentation, condensation, or abnormal morphology, while N*_total_* denotes the total number of nuclei stained with either Hoechst 33342 or Sytox Green.

Pathologically altered nuclei were assessed based on a range of morphological criteria, including chromatin condensation, reduced nucleoplasm volume, loss of the characteristic rounded shape, and fragmentation. Chromatin condensation was characterized by the dense packing of nuclear material, accompanied by increased fluorescence intensity when stained with Hoechst 33342 and Sytox Green, uneven chromatin distribution, and loss of distinct nuclear contours. These changes are typical of the early stages of apoptosis, when the cell begins to prepare for programmed death. Reduced nucleoplasm volume manifested as a decrease in nuclear diameter, dense chromatin compaction, and the formation of a more compact but structurally disrupted nuclear mass.

Nuclear fragmentation, a hallmark of the late stages of apoptosis, was identified based on several key criteria. The primary features included the formation of fully separated fragments located near the parent nucleus, significantly smaller in size. These fragments exhibited tightly packed chromatin, reflected in a pronounced fluorescence signal when stained with Hoechst 33342 and Sytox Green. The shape of these nuclear fragments varied from round to irregularly elongated, often with uneven edges and a heterogeneous internal structure, indicating a loss of nuclear integrity and the breakdown of internal architecture.

Quantitative assessment of fragmented and pathological nuclei was performed using ImageJ software (version 1.54j, National Institutes of Health, Bethesda, MD, USA), which provides high-precision manual image analysis. In addition, a custom neural network model based on YOLOv11 was used for automated recognition and counting of fragmented nuclei, serving as an additional validation tool for the manual analysis results.

### 4.5. Fluorescence Microscopy of p53 Localization

The localization of p53 in the brain 24 h after TBI was determined using immunofluorescence analysis according to the following protocol. The area surrounding the necrosis focus, caused by the impact of a weight on the skull, as well as the corresponding coordinates in the left, undamaged hemisphere, was excised. The region of interest was the parietal cortex of the brain. The excised piece of mouse brain cortex was fixed for 6 h in 4% paraformaldehyde and incubated for 48 h in 20% sucrose at 4 °C, after which it was placed on 4% agarose gel (low-melting agarose, Sigma-Aldrich, Burlington, MA, USA). Sections of agarose blocks approximately 20 μm thick were obtained using a Leica VT 1000 S vibratome (Wetzlar, Germany). After washing with PBS, the sections were incubated for 1 h at room temperature with 5% BSA and 0.3% Triton X-100 to block nonspecific binding sites. The sections were then incubated with rabbit primary antibodies against p53 (1:100; PAA928Mu01, Cloud-Clone Corp, Wuhan, China) for two days at 4 °C. Following three washes in PBS, the sections were incubated with Goat Anti-Rabbit IgG (H + L) (Cyanine3 conjugated) (1:100, Elabscience). Negative control was performed without primary antibodies. Neuronal and glial cell nuclei were visualized using Hoechst 33342 (40 µM; 10 min). The sections were mounted in 60% glycerol and examined using an Olympus BX53 fluorescence microscope (Olympus Corporation, Tokyo, Japan) equipped with a high-resolution digital camera (EXCCD01400KPA, Hangzhou ToupTek Photonics Co., Ltd., Hangzhou, China). Each group included a minimum of 6 animals.

For quantitative analysis of p53-positive cells, the proportion of these cells relative to the total number of cells was calculated and expressed as a percentage using Formula (2):

(2) $$P_{p53-positive\;cells}=\frac{N_{p53}}{N_{total}}\times 100$$

where N*_p53_* is the number of p53-positive cells, and N*_total_* is the total number of cells, determined by Hoechst 33342 nuclear staining.

Additionally, a quantitative analysis of p53-positive cells with nuclear localization of the p53 protein was performed. The count of such cells was based on the same images at ×200 magnification, using Formula (3):

(3) $$P_{p53-positive\;cell\;nuclei}=\frac{N_{p53}}{N_{total}}\times 100$$

where N*_p53_* is the number of p53-positive cells with nuclear localization of p53, and N*_total_* is the total number of p53-positive cells.

Furthermore, the percentage of p53-positive cells exhibiting signs of nuclear fragmentation was calculated using Formula (4):

(4) $$P_{fragmented\;p53-positive\;nuclei}=\frac{N_{p53}}{N_{total}}\times 100$$

where N*_p53_* is the number of p53-positive fragmented nuclei, and N*_total_* is the total number of p53-positive cells.

Fluorescence microscopy image analysis was performed using ImageJ software (version 1.54j, National Institutes of Health, Bethesda, MD, USA).

### 4.6. Hematoxylin–Eosin Staining

For histological analysis of brain tissue following TBI, sections were stained with hematoxylin (19-03, LLC “NPF BlikMedicalProduction”, Taganrog, Russia) and eosin (01-05, LLC “NPF BlikMedicalProduction”, Taganrog, Russia) (H&E). Paraffin sections were obtained using a rotary microtome (HM340E; Thermo Fisher Scientific, Walldorf, Germany). Paraffin blocks containing fixed brain tissue were pre-cooled in a refrigeration unit (HistoStar; Thermo Fisher Scientific, Walldorf, Germany) for 30 min to enhance cutting properties. Sections with a thickness of 3–4 μm were prepared using disposable blades, after initial trimming of the block (20 μm) to remove excess paraffin and expose the tissue. The sections were carefully transferred to a water bath at 42–43 °C for flattening, and then they were mounted onto glass slides and air-dried at room temperature for 24 h.

After preparation, the sections underwent routine H&E staining to visualize morphological changes in the brain tissue. The sections were first deparaffinized in three changes of xylene (ZPD-9185173, OOO “MedTechnikaPoint”, St. Petersburg, Russia), followed by rehydration in a series of alcohols with decreasing concentrations. Hematoxylin staining was performed for 25 min, after which the sections were differentiated in a 1% hydrochloric acid solution and rinsed with running water to remove excess dye. Subsequently, the sections were counterstained with eosin for 10–15 s and quickly washed with distilled water to prevent overstaining. Dehydration was carried out in alcohols of increasing concentration, followed by clearing in two changes of xylene. The sections were then mounted using a mounting medium (Vitrogel, Series: 749, Article: 12-005, LLC “Biovitrum”, St. Petersburg, Russia) and cover slips. Once dried, the slides were analyzed under a light microscope to assess structural changes, including signs of necrosis, edema, inflammation, and neuronal damage in the injury zone.

H&E staining provided clear visualization of cell nuclei and cytoplasm, enabling a detailed assessment of tissue morphology and the identification of pathological changes characteristic of TBI. Standardized staining protocols were employed to enhance accuracy and minimize variability between samples.

During the analysis of brain histological sections stained with hematoxylin and eosin, a quantitative comparison of normal and pathologically altered cells in the injury zone following TBI was conducted. Cell counting was performed at ×400 magnification across ten randomly selected fields of view. The proportion of pathological cells was calculated using Formula (5):

(5) $$P_{path}=\frac{N_{path}}{N_{total}}\times 100$$

where N*_path_* represents the number of pathologically altered cells, and N*_total_* is the total number of cells in the field of view. The calculation was performed at ×400 magnification. Pathological cells were identified based on a combination of morphological criteria, including vacuolization, karyolysis, and pyknosis, which are characteristic of irreversible cellular damage. Vacuolization is evidenced by the formation of cytoplasmic vacuoles, reflecting organelle swelling and membrane disruption. Karyolysis involves the dissolution of the nucleus due to enzymatic chromatin degradation, while pyknosis is characterized by the condensation and shrinkage of the nucleus, often accompanied by intense eosinophilic staining.

The infiltration area was measured at ×100 magnification, using Formula (6):

(6) $$Infiltration\;Area\;Percentage=\frac{Infiltrated\;Area}{Total\;area}\times 100$$

This formula calculates the percentage of the infiltration area relative to the total tissue area. “Infiltrated Area” refers to the measured area of inflammatory infiltration, identified based on the presence of dense clusters of leukocytes, as well as erythrocytes indicating vascular damage. The “Total Area” is the entire area of the examined section. The resulting value, expressed as a percentage, provides a quantitative assessment of the extent of the inflammatory process and reflects the degree of secondary damage following TBI. All image analyses were performed using ImageJ software (version 1.54j; National Institutes of Health, Bethesda, MD, USA).

### 4.7. Molecular Dynamics Simulation of p53 Complexes with H_2_S Derivatives

To investigate the potential binding of H_2_S molecules to the p53 protein, molecular dynamics (MD) simulations were conducted, followed by an analysis of the average binding energy. Variants of the p53 protein from human (*Homo sapiens*, P04637), fruit fly (*Drosophila melanogaster*, Q8IMZ4), zebrafish (*Danio rerio*, P79734), and chicken (*Gallus gallus*, P10360) were selected as protein models, sourced from the Uniprot database. Full protein models predicted by AlphaFold 2.0 (DeepMind, London, United Kingdom) [53] were used. Preparation of the proteins in a water-ion environment, as well as the necessary structure and charges of the H_2_S molecule, was performed using the standard utilities of the AmberTools24 software package [54]. Molecular dynamics simulations were carried out using the following force fields:

- Amber FF19SB [55]—for modeling p53;
- TIP3P—for modeling water molecules and the ionic environment;
- GAFF2 [56]—for modeling hydrogen sulfide derivatives.

Two sets of four models were generated: a reference set without the hydrogen sulfide molecule and an experimental set with one H_2_S molecule added to each model. The simulation was performed using the GROMACS software (version 2024.3; KTH Royal Institute of Technology, Stockholm, Sweden) in eight stages: steepest descent minimization → l-bfgs minimization till to 100 kcal/mol energy minimization tolerance → five stages of equilibration in the NPT ensemble (constant number of particles, pressure, and temperature) → MD production. The MD simulation ran for 100 ns at a temperature of 310 K and an ionic concentration of 0.14 M K^+^ and Cl^−^. The system’s charge was neutralized with additional K^+^ or Cl^−^ ions. Differences in RMSD (root-mean-square deviation) and radius of gyration plots were analyzed, and an MMPBSA (Molecular Mechanics Poisson–Boltzmann Surface Area) analysis [57] was conducted to assess the binding of hydrogen sulfide to the p53 protein variants.

Additionally, simulations were performed with human p53 in four configurations: neutral complexes with H_2_S and HS^−^, protonated p53 with H_2_S, and protonated mutant p53 (R248Q) obtained using the docking utility AutoDock Vina version 1.2.5 (Scripps Research Institute, La Jolla, CA, USA) [58].

The docking results were visualized using UCSF Chimera version 1.17.3 (University of California, San Francisco, CA, USA).

### 4.8. Bioinformatics Analysis

For multiple alignment of amino acid sequences, the Clustal Omega method was utilized, accessible through the web interface of the European Bioinformatics Institute (EBI) (www.ebi.ac.uk/Tools/msa/clustalo; accessed on 17 March 2025). This method, based on a progressive alignment algorithm, is optimized for accurate and scalable analysis of large datasets. Amino acid sequences were retrieved from the Uniprot database. The protein database, containing sequences from various species, was used for the search. Sequence selection was based on their annotations and relevance to the study’s objectives. Alignment was performed using the Clustal Omega tool on the EBI platform, accessible via the “Run Clustal Omega” link. Default parameters of the method were applied to ensure high alignment accuracy. This approach enabled the identification of conserved and variable regions in the amino acid sequences, which is critical for analyzing the evolutionary and functional aspects of proteins.

Following the multiple alignment, an identity matrix was constructed—a table in which the percentage of amino acid identity was calculated for each pair of sequences. Identity reflects the proportion of matching amino acids in the aligned sequences and serves as a key indicator for assessing the degree of evolutionary relatedness between proteins. The identity matrix was generated based on the alignment results for all protein pairs included in the study. Each element of the matrix corresponds to the percentage of amino acid matches for a specific pair of sequences. High identity values (above 30%) suggest potential protein homology, supporting a shared evolutionary origin. Low values (below 25%) may indicate a distant evolutionary relationship or functional differences. The matrix facilitates the visualization of evolutionary relationships between proteins from different organisms, highlighting conserved regions and potential functional changes. The analysis was conducted for the p53 protein across representatives of various species and classes (Table 5).

The obtained multiple alignments were analyzed to identify conserved domains significant for functional and structural studies, as well as for constructing phylogenetic trees to examine the evolutionary history of proteins.

### 4.9. Optimization of YOLO Models for Nuclei Detection in Biological Imagery

This study evaluated and optimized YOLO models (versions 8 and 11, Ultralytics, United States) for object detection in biological imagery, with the primary objective of enhancing model performance, as assessed by the F1-score metric, while addressing challenges posed by the underrepresented apoptosis class. Model training leveraged internal YOLO mechanisms, where improvement was determined by the mAP@0.5 metric (mean average precision at an IoU threshold of 0.5). Final model comparisons were based on F1-score post-training. The training dataset comprised 37 images, and 2 classes (normal and apoptotic nucleus) containing approximately 3200 labeled nuclei, providing a focused yet challenging set for model optimization.

An object detection model based on YOLO was developed to classify nuclear states—apoptotic vs. normal—in fluorescence microscopy images. Annotations were performed by a single annotator, using Roboflow (https://roboflow.com/, https://app.roboflow.com/cell-qttak/fluorescent-cell/3; accessed on 22 May 2025) in object detection mode, selected for its ease, speed, and sufficient precision. Criteria for classification were established as follows: normal nuclei were defined by well-defined, non-blurred edges with homogeneous chromatin staining, while apoptotic nuclei were identified by the presence of chromatin condensation and fragmentation, often manifesting as “bubbles” or irregular, bleb-like structures. Contrast and shadows were maximized, and brightness was adjusted to a level where at least a few objects remained discernible. Images exhibiting the most significant visual deviations were allocated to the validation set. This binary distinction was consistently applied across all annotations to minimize subjectivity.

Images were initially provided in RGB format and processed using baseline hyperparameters for YOLO 8 and YOLO 11, tailored to biological data. The baseline configuration in the train.py script included 1000 epochs, a batch size of 2, and image dimensions of 640 × 640. Training was conducted on an Nvidia 4090 GPU, providing the computational power needed for a 1000-epoch schedule. This extended epoch count was chosen to allow for exploration of the loss landscape given the small dataset size, with early stopping implemented at 50 epochs of no improvement in validation loss to prevent overfitting. Input images were resized to 640 × 640 pixels. The AdamW optimizer was selected for its balance of convergence speed and stability, with an initial learning rate of 0.002—determined via a preliminary grid search (0.0001–0.01) to maximize gradient descent efficiency—and a cosine decay schedule reducing to 0.01 over training. Weight decay was set to 0.0005 to regularize the model, a value standard for YOLO architectures.

Augmentations with parameters were presented by hsv_h = 0.015, hsv_s = 0.7, hsv_v = 0.4, degrees = 10.0, translate = 0.1, scale = 0.5, shear = 2.0, perspective = 0.0005, fliplr = 0.5, mixup = 0.2, and copy_paste = 0.1. Mosaic augmentation was set to 1.0, with mosaics disabled for the final 10 epochs (close_mosaic = 10). To address the underrepresented apoptosis class, class weights of [4.0, 1.0] were introduced in the data.yaml file to enhance its prominence during training.

The experimental workflow began with baseline training using mosaic images and standard YOLO augmentations for both YOLO 8 and YOLO 11. Subsequently, class weights [4.0, 1.0] were applied to both model versions, and their impact on F1-score was assessed. Following evaluation, weights were removed due to observed metric degradation. Preprocessing steps were then implemented via Roboflow, converting RGB images to grayscale and introducing three augmentations (horizontal flip, vertical flip, and 20% zoom), while disabling mosaic and YOLO-native augmentations. Finally, mosaic and YOLO augmentations were reinstated to evaluate their effect on performance. Seven configurations were tested. For each, both the iteration internally yielding the highest mAP@0.5 (best.pt) and the corresponding F1-score were recorded. Weights were exported in ONNX format.

The evaluation centered on several critical performance indicators. The mAP50, defined as the mean average precision at an IoU threshold of 0.5, quantifies detection accuracy by requiring a minimum of 50% overlap between the predicted and ground-truth bounding boxes. Additionally, the mAP50-95, which represents the mean average precision averaged over a range of IoU thresholds from 0.5 to 0.95 in 0.05 increments, offers a more comprehensive assessment of the model’s robustness across varying degrees of overlap. Sensitivity was further evaluated through the recall metric, which measures the proportion of true-positive detections, thereby providing insight into the model’s capacity to identify cells with low-contrast signals or faint fluorescence. Additionally, model performance was assessed using precision and sensitivity metrics, offering a more nuanced evaluation of classification accuracy, particularly in distinguishing apoptotic from normal nuclei under varying image-quality conditions. Finally, the classification loss, associated with the model’s ability to accurately classify neural cell subtypes, reflects its overall performance in distinguishing among different cellular populations.

To enable real-time inference and user accessibility, the optimized YOLO models were integrated into a Telegram-based interface using the Python telegram library version 20.7 and telegram.ext libraries. A YOLO-based image-processing pipeline was implemented using a custom handler, which received and processed incoming images, applied ONNX-exported model inference via the ultralytics library, and returned bounding-box annotated images alongside class-wise detection counts.

Detection output included both annotated images and per-class object counts, re-turned to the user within the same chat thread. This interaction model was made possible via asynchronous message handling and image-annotation rendering, offering a low-latency experience.

A Telegram-based deployment was implemented, leveraging long polling for minimal server load and secure data transmission. Telegram was selected for its inherent security and cross-platform compatibility, simplifying development and ensuring user data privacy.

### 4.10. Statistical Analysis

Statistical analysis was performed using one-way analysis of variance (ANOVA) with Tukey’s post hoc test. Normality and homogeneity of variance were assessed using the Shapiro–Wilk test and the Brown–Forsythe test, respectively. If normality or homogeneity of variance was not confirmed, the non-parametric Kruskal–Wallis test was applied. All study results were analyzed blindly. Differences were considered significant at *p* < 0.05 and n = 5. The obtained data were expressed as mean ± standard deviation. Statistical analysis was conducted using the software packages SigmaPlot version 12.5 (Systat Software Inc., San Jose, CA, USA) and JASP version 0.19.1 (University of Amsterdam, Amsterdam, Netherlands).

## 5. Conclusions

The results of the present study demonstrate that the intracellular localization of p53 and its expression level are closely associated with the nature of cell death in the damaged cerebral cortex following TBI. The use of three-dimensional confocal imaging enabled the identification of significant differences in p53 distribution between intact and injured brain regions, as well as among different cell types. In neurons, cytoplasmic expression of p53 predominated, which was associated with activation of the mitochondrial apoptotic pathway, whereas in astrocytes, nuclear accumulation of the protein was observed, indicating the initiation of a transcription-dependent cell death cascade.

The correlation between nuclear localization of p53 and morphological signs of late-stage apoptosis, such as nuclear fragmentation and apoptotic body formation, supports the active role of this protein in the pathogenesis of traumatic brain injury. Pharmacological modulation of H_2_S levels significantly affected p53 expression and the extent of apoptosis: administration of Na_2_S_2_O_3_ reduced nuclear p53 localization and the number of apoptotic cells, whereas inhibition of H_2_S biosynthesis with AOAA produced the opposite effect. These findings highlight the significant regulatory role of H_2_S in modulating p53-dependent signaling pathways.

Additionally, evolutionary and molecular dynamics analyses confirmed the high conservation of p53 among vertebrates and revealed the potential of H_2_S—particularly in its neutral form—to modulate its conformational dynamics. This opens new avenues for investigating H_2_S as a regulator of the structural flexibility and functional activity of p53, especially under conditions of oxidative stress and pH imbalance following TBI.

The application of the YOLO model for automated analysis of fluorescence microscopy images enabled quantitative assessment of nuclear fragmentation patterns across experimental groups. The YOLOv8 model demonstrated high localization accuracy (mAP50 = 0.727), with optimal F1-scores reaching 45.1% under specific preprocessing settings, while YOLOv11 exhibited higher sensitivity (F1 up to 62.7%) when grayscale conversion and extended augmentation techniques were applied. The implementation of a Telegram bot based on YOLO allowed for efficient and reproducible real-time cell classification, enhancing the potential for automated morphometric analysis in neurobiological research.

Taken together, these findings emphasize the pivotal role of p53 as a molecular mediator linking injury, metabolic stress, and cell death, and highlight the significant neuroprotective potential of H_2_S, which may be leveraged in the development of novel therapeutic strategies for TBI. The integration of computer vision approaches opens new possibilities for standardizing morphological assessments and improving the accuracy of quantitative analyses in neurodegeneration studies.

## Figures and Tables

**Table 1:** MMPBSA calculation results (total delta values). All values are given in kcal/mol.

Protein	Generalized Born			Poisson–Boltzmann		
	Average	Std. Dev.	Std. Err. of Mean	Average	Std. Dev.	Std. Err. of Mean
1st experiment: Screening with H_2_S						
Chicken	0.0253	0.1344	0.0190	0.6076	0.3437	0.0486
Drosophila	−0.2103	0.8083	0.1143	0.6509	0.3979	0.0563
Human	0.0221	0.1406	0.0199	0.6395	0.2321	0.0328
ZebraFish	−0.0357	0.2775	0.0392	0.5310	0.2981	0.0422
2nd experiment: Human p53 with variant of H_2_S						
Human + H_2_S	−1.1648	2.1206	0.3353	−0.3828	3.1760	0.5022
Human + HS^−^	0.0784	1.5935	0.2520	0.1866	2.4618	0.3892
Human (prot) + H_2_S	−0.0845	0.6624	0.0937	0.6429	0.3373	0.0477
Human (mut) + H_2_S	−0.2378	0.8503	0.0380	0.5912	0.9736	0.0435

**Table 2:** Percent identity matrix for p53—generated by Clustal2.1.

No.	Species	1	2	3	4	5	6	7	8	9	10
1	*Mus musculus*	100.00	78.04	75.00	49.86	47.84	50.00	52.15	46.27	21.22	20.51
2	*Homo sapiens*	78.04	100.00	81.56	50.56	49.87	52.36	55.40	48.22	21.16	20.90
3	*Bos taurus*	75.00	81.56	100.00	51.86	51.77	51.52	54.34	47.31	21.30	21.97
4	*Danio rerio*	49.86	50.56	51.86	100.00	60.11	55.26	55.10	51.05	21.74	19.94
5	*Erpetoichthys calabaricus*	47.84	49.87	51.77	60.11	100.00	54.13	53.67	46.59	22.12	20.23
6	*Sphenodon punctatus*	50.00	52.36	51.52	55.26	54.13	100.00	61.51	56.46	21.74	21.18
7	*Gallus gallus*	52.15	55.40	54.34	55.10	53.67	61.51	100.00	65.60	24.15	22.29
8	*Catharus ustulatus*	46.27	48.22	47.31	51.05	46.59	56.46	65.60	100.00	22.26	21.63
9	*Drosophila melanogaster*	21.22	21.16	21.30	21.74	22.12	21.74	24.15	22.26	100.00	36.27
10	*Bactrocera dorsalis*	20.51	20.90	21.97	19.94	20.23	21.18	22.29	21.63	36.27	100.00

**Table 3:** Comparative analysis of models’ efficacy.

YOLO Version	F1-Score	Sensitivity	Precision	Specificity	mAP@0.5	mAP@0.5:0.95
8	45.1%	52.1%	39.7%	63.7%	72.7%	17.7%
8	38.6%	49.5%	31.6%	57.9%	69.3%	15.8%
11	50.5%	64.1%	41.5%	71.2%	60.2%	11.2%
11	34.1%	39.8%	29.9%	54.2%	57.0%	10.0%
11	41.2%	43.2%	39.3%	72.2%	59.0%	10.5%
11	34.5%	40.9%	31.3%	54.6%	52.0%	8.5%
11	62.7%	82.0%	52.8%	77.1%	61.5%	11.9%

**Table 4:** Table with comparison of preprocessing methodics and hyperparameters.

YOLO Version	Best.pt’s Epoch	F1-Score	Grayscale Images	Image Augmentations	YOLO Augmentations	YOLO Mosaic Images for Train	Enhanced Weights for Underrepresented Class
8	225	45.1%	-	-	+	+	-
8	200	38.6%	-	-	+	+	+
11	317	50.5%	-	-	+	+	-
11	208	34.1%	-	-	+	+	+
11	214	41.2%	-	+	+	+	-
11	261	34.5%	+	+	-	-	-
11	350	62.7%	+	+	+	+	-

**Table 5:** Amino acid sequences of p53 from different organisms were used for multiple alignment to assess the evolutionary conservation of this protein.

Class	Animal Species	Sequence Identifier	Number of Amino Acid Residues
Insects	*Drosophila melanogaster*	Q8IMZ4	495
	*Bactrocera dorsalis*	A0A034VRW3	507
Fish	*Danio rerio* (*Zebrafish*)	P79734	373
	*Erpetoichthys calabaricus*	A0A8C4SF19	395
Birds	*Gallus gallus*	P10360	367
	*Catharus ustulatus*	A0A8C3UX00	352
Reptiles	*Sphenodon punctatus*	A0A8D0L9L5	317 AA
Mammals	*Homo sapiens*	P04637	393
	*Mus musculus*	P02340	390
	*Bos taurus*	P67939	386

## Data Availability

The original contributions presented in this study are included in the article/Supplementary Materials. Further inquiries can be directed to the corresponding author.

## Supplementary Materials

The following supporting information can be downloaded at https://www.mdpi.com/article/10.3390/ijms26115066/s1.
